# ALAS2 Prevents Neonatal Necrotizing Enterocolitis by Improving Ferroptosis in Intestinal Epithelial Cells Through Inhibition of Oxidative Stress

**DOI:** 10.1155/mi/6683001

**Published:** 2026-01-07

**Authors:** Zenghui Hao, Jinbao Han, Ting Yao, Zheng Zhao, Wei Fan, Zaiqun Jiang, Yunting Wang, Xiaoqian Yang, Zhilin Xu

**Affiliations:** ^1^ Department of Pediatric Surgery, The Sixth Affiliated Hospital of Harbin Medical University, Harbin, China; ^2^ Department of Emergency Center, Beijing Children’s Hospital, Capital Medical University, National Center for Children’s Health, Beijing, China, ccmu.edu.cn

**Keywords:** ALAS2, ferroptosis, neonatal necrotizing enterocolitis, oxidative stress

## Abstract

Neonatal necrotizing enterocolitis (NEC) is an intestinal disease that occurs in the neonatal period. The purpose of this study was to investigate the role of 5′‐aminolevulinate synthase 2 (ALAS2) in NEC‐induced intestinal injury. In a neonatal mouse, NEC model was induced by high‐osmolarity formula and hypoxia‐cold stress, and ALAS2 expression was significantly downregulated in ileal tissues (*p*  < 0.01), coinciding with elevated oxidative stress (increased Fe^2+^/malondialdehyde [MDA] and decreased superoxide dismutase [SOD]), inflammation (increased TNF‐α/interferon‐gamma [IFN‐γ]), and ferroptosis activation (increased acyl‐CoA synthetase long‐chain family member 4 [ACSL4] and decreased ferritin heavy chain 1 [FTH1] with mitochondrial shrinkage). In vitro, tumor necrosis factor‐alpha (TNF‐α)/IFN‐γ‐treated intestinal epithelial cell (IEC) exhibited progressive ALAS2 suppression and increased necrosis. Crucially, lentivirus‐mediated ALAS2 overexpression reversed these effects, reducing cell necrosis by 22% while suppressing ferroptosis markers (Fe^2+^ accumulation, lipid reactive oxygen species [ROS], and mitochondrial depolarization) and oxidative damage (decreased MDA and restored glutathione [GSH]/catalase [CAT]/SOD). Untargeted metabolomics further revealed ALAS2‐mediated modulation of nutrient metabolism and redox pathways. Collectively, ALAS2 ameliorates NEC by blocking oxidative stress‐driven ferroptosis in IECs, proposing a novel therapeutic target.

## 1. Introduction

Neonatal necrotizing enterocolitis (NEC) remains a devastating gastrointestinal emergency predominantly affecting premature infants, with an incidence ranging from 5% to 10% in very low birth weight infants and associated mortality rates reaching 20%–30% [1]. Despite advances in neonatal care, NEC continues to be a leading cause of morbidity and mortality in neonatal intensive care units worldwide [2]. This disease is characterized by intestinal inflammation, barrier disruption, and, ultimately, necrosis of the bowel wall [3, 4]. The pathogenesis of NEC is multifactorial and incompletely understood, involving an intricate interplay between prematurity, intestinal dysbiosis, ischemia–reperfusion injury, and a dysregulated host immune response within the immature gut [5, 6]. A critical consequence of these insults is the excessive activation of inflammatory cascades and the generation of oxidative stress, which are central drivers of intestinal epithelial cell (IEC) damage and the progression to tissue necrosis [7]. The in‐depth exploration of the pathogenesis of NEC could help to further improve the diagnosis and treatment of the disease.

Inflammation is a hallmark of NEC pathophysiology. Pro‐inflammatory cytokines, such as tumor necrosis factor‐alpha (TNF‐α) and interferon‐gamma (IFN‐γ), are significantly elevated in NEC and play pivotal roles in amplifying the inflammatory response and disrupting the intestinal barrier, thereby creating a vicious cycle of injury [8–10]. Concurrently, oxidative stress, resulting from an imbalance between the production of reactive oxygen species (ROS) and the capacity of antioxidant defense systems, is a key contributor to NEC development [11]. The immature intestine of preterm infants is particularly vulnerable to oxidative damage due to underdeveloped antioxidant mechanisms [12]. Oxidative stress directly damages cellular components and is closely linked to other forms of regulated cell death (RCD) [13, 14]. Among RCD pathways, ferroptosis has emerged as a significant mechanism in various gastrointestinal pathologies, including inflammatory bowel disease and ischemia–reperfusion injury [7, 15]. Ferroptosis is an iron‐dependent form of RCD driven by the accumulation of lipid peroxides, stemming from the disruption of cellular antioxidant defenses and alterations in iron metabolism [16]. Proteins such as acyl‐CoA synthetase long‐chain amily member 4 (ACSL4) promote ferroptosis by facilitating the incorporation of polyunsaturated fatty acids into membrane phospholipids, while ferritin heavy chain 1 (FTH1) plays a protective role by sequestering iron [17, 18]. Overall, the convergence of inflammation and oxidative stress creates an environment conducive to ferroptosis in IECs, potentially representing a critical, yet underexplored, axis in NEC pathogenesis.

5′‐Aminolevulinate synthase 2 (ALAS2), the rate‐limiting enzyme in the heme biosynthesis pathway within erythroid cells, is crucial for iron utilization and hemoglobin production [19–21]. ALAS2 activity influences cellular iron metabolism and redox homeostasis, processes intrinsically linked to ferroptosis susceptibility [22, 23]. However, the specific role of ALAS2 in intestinal inflammation, particularly in the context of NEC and its associated cell death pathways like ferroptosis, has not been investigated. By analyzing the original data from the GEO databases (GSE64801, GSE193177, and GSE198372), we found that ALAS2 expression was significantly downregulated in the ileum tissues of acute premature NEC patients and neonatal mice in the NEC model. Based on the above studies, it is reasonable to hypothesize that ALAS2 may be involved in the progression of NEC and is likely to be associated with ferroptosis.

The aim of this study was to establish models of NEC in vivo and in vitro to investigate the effect of ALAS2 on NEC. ALAS2 improved NEC by inhibiting oxidative stress and ferroptosis in IECs. The results of this study may provide useful information on the theoretical basis of NEC therapy.

## 2. Materials and Methods

### 2.1. Animal Models

The neonatal NEC mouse model was performed as previously described, with some adjustments [24, 25]. Ten‐week‐old male and female C57BL/6 mice were purchased from Huachuang Sino (China) and produced by free mating and pregnancy. At postnatal Day 10 (P10), newborn mice were randomly divided into two groups: control and neonatal NEC groups. Mice in the control group were breastfed without any intervention. Mice in the NEC group were separated from their mothers and reared in a constant temperature box at 28°C. At postnatal Day 10–14 (P10‐14), mice were then given formula milk at 30 µL/g body weight via oral gavage every 4 h, and neonatal mice were subjected to hypoxic stimulation (5% oxygen and 95% nitrogen) for 2 min and cold stimulation (4°C) for 10 min twice a day. Body weight and survival condition were recorded once daily. After the experiment, intestinal tissues were collected for subsequent experiments. All procedures involving animal experiments were performed with consent from the Ethics Committee of the Sixth Affiliated Hospital of Harbin Medical University (Approval Number LC2024‐078).

### 2.2. Cell Lines and Kits

Rat IEC‐6 cells were purchased from iCell Bioscience Technology Co., Ltd. (China). IEC‐6 cells were cultured with DMEM (Servicebio, China) containing 10% FBS (Tianhangbio, China), 1% antibiotics (20 μg/mL penicillin [Solarbio, China] and 100 μg/mL streptomycin [Solarbio, China]), and 0.01 mg/mL insulin (Biosharp, China). Cells were maintained in a 37°C, 5% CO_2_ incubator (Heal Force, China).

The content of TNF‐α and IFN‐γ was detected by the Mouse TNF‐α ELISA Kit (Liankebio, China) and Mouse IFN‐γ ELISA Kit (Liankebio, China), respectively. The content of iron ions in tissues and cells were determined by the Ferrous Iron Colorimetric Assay Kit (Elabscience, China) and Cell Ferrous Iron Colorimetric Assay Kit (Elabscience, China), respectively. The Malondialdehyde (MDA) Assay Kit, Superoxide Dismutase (SOD) Assay Kit, Reduced Glutathione (GSH) Assay Kit, and Catalase (CAT) Assay Kit were purchased from Nanjing Jiancheng (China).

### 2.3. Lentivirus Infection

The ALAS2 coding sequence (NM_013197) was inserted into the XhoI/NotI site of the lentivirus expression vector pLVX‐IRES‐puro, which was then mixed thoroughly with the envelope and packaging plasmids (pMD2.G and pSPAX2). Then, HEK293T cells were cotransfected with Lipo 3000 (Invitrogen, USA) according to the instructions of the supplier to produce lentivirus. IEC‐6 cells were inoculated on 6‐well plates, and when 50% fusion was achieved, the cells were infected with lentivirus at a multiplicity of infection (MOI) of 50 and further cultured at 37°C in a 5% CO_2_ incubator.

### 2.4. Hematoxylin and Eosin (H&E) Staining

Embedded tissues were dewaxed using xylene and dehydrated in gradient ethanol. Sections were stained with hematoxylin (Solarbio, China) for 5 min, and then soaked in distilled water for 5 min. Then, they were stained with eosin (Sangon, China) for 3 min. Finally, sections were dehydrated with graded alcohol, transparented with xylene, and coverslipped with resin.

### 2.5. Immunofluorescence

The 5‐μm paraffin‐embedded tissue sections were dehydrated with graded ethanol, washed with PBS, and blocked with BSA (Sangon, China) for 15 min. Sections were incubated with primary anti‐ALAS2 antibody (cat# PA5‐143885, 1:200, Thermo Fisher, USA) at 4°C overnight and then sequentially incubated with fluorescent secondary antibody (cat# 4413, 1:200, CST, USA) for 1 h and DAPI (Aladdin, China). Finally, sections were visualized and photographed under a fluorescence microscope.

### 2.6. Reverse Transcription Quantitative PCR (RT‐qPCR) Analysis

Total RNA was extracted from the tissue or cells using a TRIzol reagent (BioTeke, China), and then the concentration of RNA was determined by an ultraviolet spectrophotometer (Thermo, USA). The All‐in‐One First‐Strand SuperMix Kit (Magen, China) was used to reverse transcribe RNA to cDNA according to the manufacturer’s instructions. The PCR reaction was performed using the SYBR Green Kit (Solarbio, China), and RNA levels were quantified by the Exicycler 96 fluorescence quantifier (Bioneer, Korea). The relative expression levels of the genes were determined against β‐actin levels in the samples. The primers were designed as follows: mus ALAS2 F, 5′‐GCCAGGCTTTCGGTTAT‐3′, ALAS2 R, 5′‐CGAGGGTGTCTGCTTAT‐3′; rat ALAS2 F, 5′‐GACTCGGCTCTGCTCTT‐3′, ALAS2 R, 5′‐GTTACGAATGCCTTGGA‐3′.

### 2.7. Western Blot Analysis

Cells or tissues were harvested and lysed in the lysate (Beyotime, China) at 1000 × *g* at 4°C for 5 min to collect supernatants. The protein content was measured by the BCA Protein Assay (Beyotime, China). The total protein sample was then electrophoresed through sodium dodecyl sulfate–polyacrylamide gel electrophoresis (Beyotime, China) and transferred to polyvinylidene fluoride(PVDF) membranes (Abcam, UK). The membranes were blocked with the sealing solution (Beyotime, China) at room temperature for 1 h and subsequently incubated with primary antibodies at 4°C overnight. The next day, after incubation with secondary antibodies directed against goat anti‐rabbit (cat# A0208, 1:5000, Beyotime, China) or goat anti‐mouse (cat# A0216, 1:5000, Beyotime, China) at 37°C for 45 min, the PVDF membrane was developed by the ECL luminol (Beyotime, China), and Gel‐Pro‐Analyzer software was used for quantification. The following antibodies were used in the test: ALAS2 antibody (cat# PA5‐143885, 1:1000, Thermofisher, China), ACSL4 antibody (cat# sc‐271800, 1:500, Santa Cruz, China), and FTH1 antibody (cat# PA5‐27500, 1:1000, Thermofisher, China).

### 2.8. Lipid ROS Measurement

Relative lipid ROS levels in cells were assessed by the C11 BODIPY 581/591 lipid peroxidation fluorescent probe (Maokangbio, China). Cells were washed with PBS and incubated with 10 μM C11 BODIPY 581/591 probe reserve solution for 30 min at 37°C. Images were acquired and analyzed using a fluorescence microscope.

### 2.9. Flow Cytometry

Briefly, cells were centrifuged at 150 ×*g* for 5 min to remove the supernatant and then collected. Cells were then washed twice with PBS and resuspended with 500 μL binding buffer. Five microliters of annexin V‐fluorescein isothiocyanate and 5 μL of propidium iodide (PI) were added sequentially (KeyGen, China). The samples were incubated at room temperature for 10 min away from light, and the cell necrosis was detected by a flow cytometry (Agilent, USA).

### 2.10. Cell Counting Kit‐8 (CCK‐8) Assay

Cell viability was measured using the CCK‐8 Assay Kit (KeyGen, China) according to the manufacturer’s instructions. Cells at a density of 5 × 10^3^ cells/well were seeded in 96‐well plates and cultured in 37°C, 5% CO_2_ incubator. Ten microliters of CCK‐8 solution was added to each well and incubated for 2 h. The absorbance was measured at 450 nm using an automated microplate reader (BIOTEK, USA).

### 2.11. JC‐1 Staining

To monitor the alterations in mitochondrial membrane potential, the JC‐1 Apoptosis Detection Kit (KeyGen, China) was applied to IEC‐6 cells. In brief, after cells were cleaned with PBS, 1 mL of JC‐1 staining solution was added, and cells were then incubated at 37°C without light for 15–20 min. Finally, cells were washed twice with 1 × Incubation Buffer and examined under the fluorescence microscope (OLYMPUS, Japan).

### 2.12. Statistical Analysis

All experimental data are presented as the mean ± SD. GraphPad Prism 9.5 software was used to analyze the data. The unpaired *t*‐test was used to evaluate the significance of differences between the two experimental groups. The comparison among multiple groups was performed by one‐way ANOVA followed by Tukey’s post hoc test. *p*  < 0.05 was considered statistically significant.

## 3. Results

### 3.1. The Differential Expression Genes of NEC Were Presented in Different Databases

The search for effective biomarkers of neonatal NEC is of great importance for the diagnosis and treatment of the disease. The original data of normal and neonatal NEC tissues were obtained from the GEO dataset (https://www.ncbi.nlm.nih.gov/), and three datasets (GSE64801, GSE193177, and GSE198372) were selected. The differentially expressed genes (DEGs) in the intestinal tissues of three NEC‐related datasets were further analyzed, and DEGs were selected by |log_2_FC| > 1.5 and adj. *p* < 0.05. As shown in Figure 1a, the heatmap showed the distribution of these DEGs, and the specific genes were presented in Tables S1–S3. In addition, the Venn analysis of three datasets indicated that ALAS2 was the only gene shared by all three datasets (Figure 1b). ALAS2 plays a role in a variety of diseases, but the association with NEC has not been reported. Therefore, we chose ALAS2 for in‐depth study.

Figure 1The differential expression genes of neonatal necrotizing enterocolitis (NEC) were presented in different databases. (a) Heatmap of DEGs in intestinal tissue of control and NEC group in NEC datasets GSE198372, GSE193177, and GSE64801. DEGs were selected by |log_2_FC| > 1.5 and adj. *p* < 0.05. (b) The Venn diagram illustrated a total of 1 downregulated gene in the three datasets. DEGs, differentially expressed genes; FC, fold change.

**Figure:**
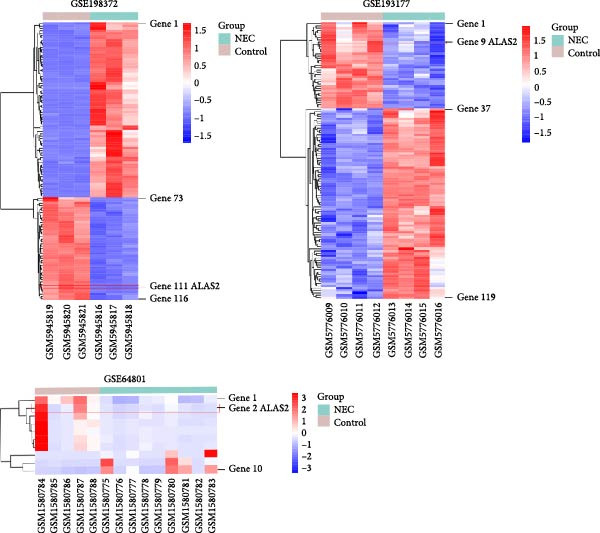
(a)

**Figure:**
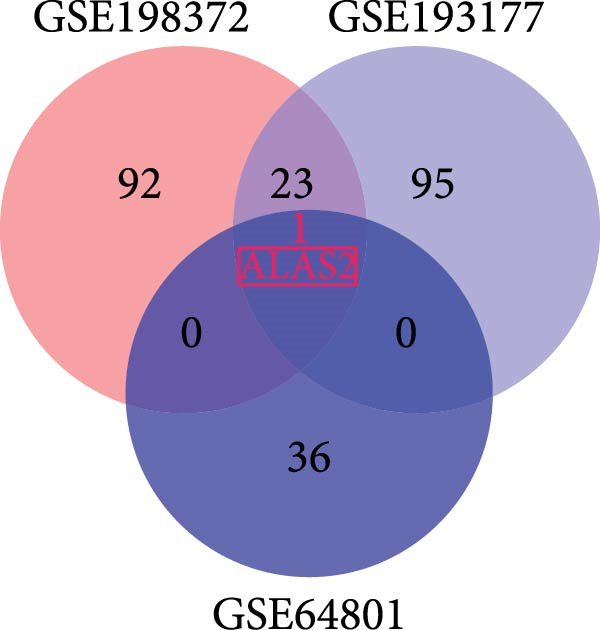
(b)

### 3.2. Establishment of Neonatal Mice NEC Model

The neonatal mice NEC model was established, and the body weight and death of the animals were recorded every day from the postnatal Day 10 (P10) to postnatal Day 14 (P14). As shown in Figure 2a, there was no mortality in the control group and 20% mortality in the NEC group. During the modeling period, the body weight of mice in the NEC group decreased significantly (Figure 2b). Compared with the control group, significant intestinal swelling, intestinal gas deposition, and thinning of the intestinal wall were observed in the NEC group (Figure 2c). In addition, assessment of intestinal tissue appearance showed dilatation of intestinal lumen and color change of the intestinal wall in the NEC group, whereas no significant intestinal damage was seen in the control group (Figure 2d). The intestinal histopathology changes were observed by H&E staining, and the intestinal tissues of the control group were arranged in an orderly manner with complete structure, while the intestinal tissues of the NEC group were arranged irregularly with thin muscle layer and severe necrosis (Figure 2e). The contents of TNF‐α and IFN‐γ in intestinal tissues of NEC group were also significantly higher than those of control group (Figure 2f). In summary, the NEC model was successfully established in neonatal mice.

Figure 2Establishment of neonatal mice NEC model. (a) Survival curves of neonatal mice in the control (*n* = 8) and NEC groups (*n* = 12). (b) Body weight changes of neonatal mice in two groups. (c) Gross morphology of the intestine tissues of neonatal mice in two groups. (d) Morphological observations of intestinal tissue in two groups. (e) H&E staining images of intestine tissues in two groups. Original magnification: ×40 and × 100; scale bar, 500 μm and 200 μm. (f–g) The expression of TNF‐α and IFN‐γ in the intestinal tissue of each group was detected by ELISA. Data are presented as mean ± SD. ELISA, enzyme‐linked immunosorbent assay.  ^∗^ *p* < 0.05,  ^∗∗^ *p* < 0.01.

**Figure:**
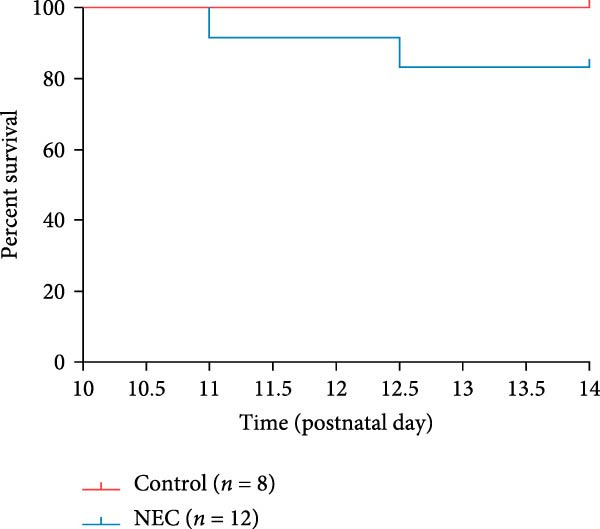
(a)

**Figure:**
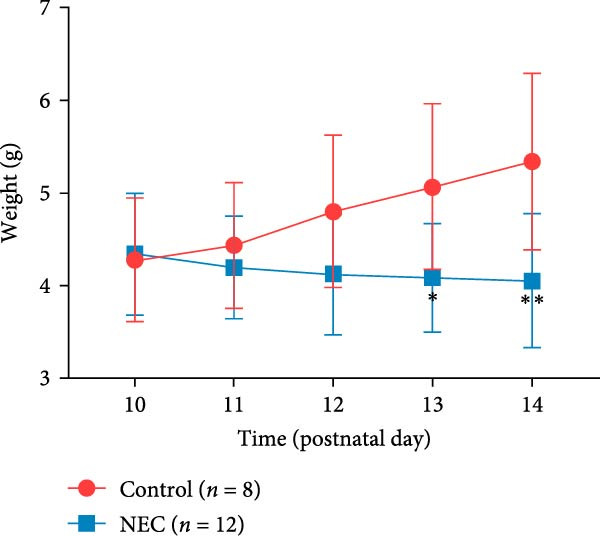
(b)

**Figure:**
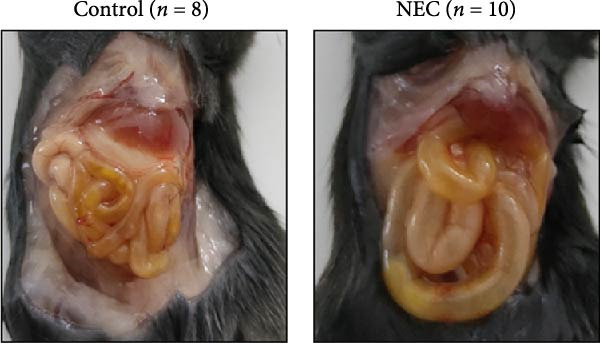
(c)

**Figure:**
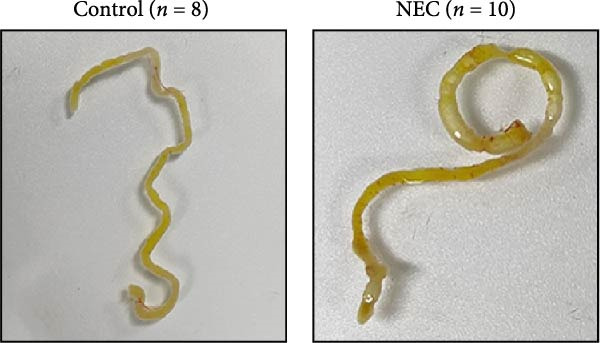
(d)

**Figure:**
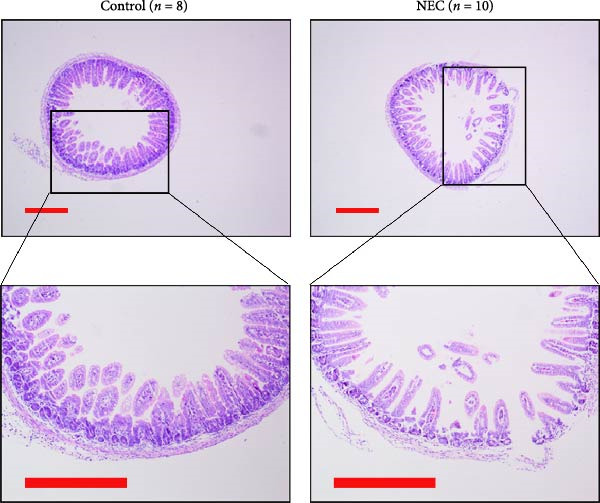
(e)

**Figure:**
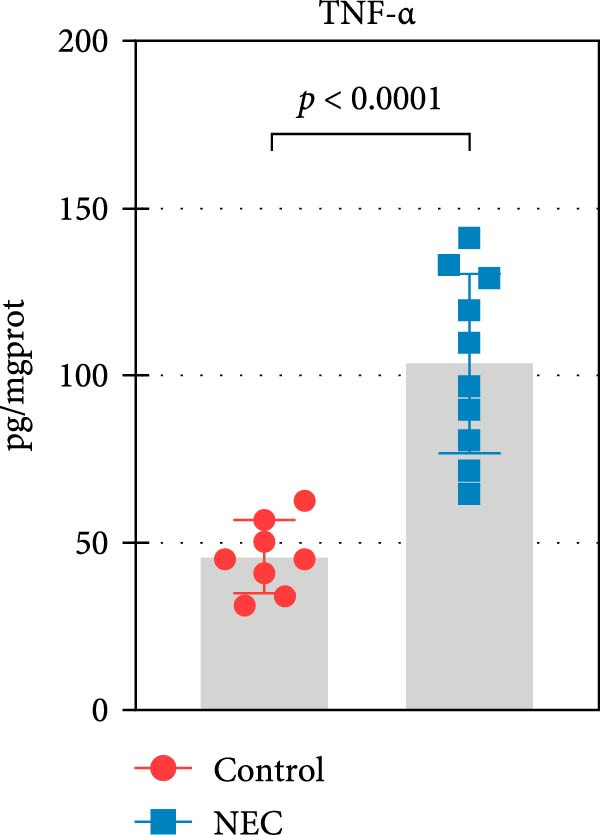
(f)

**Figure:**
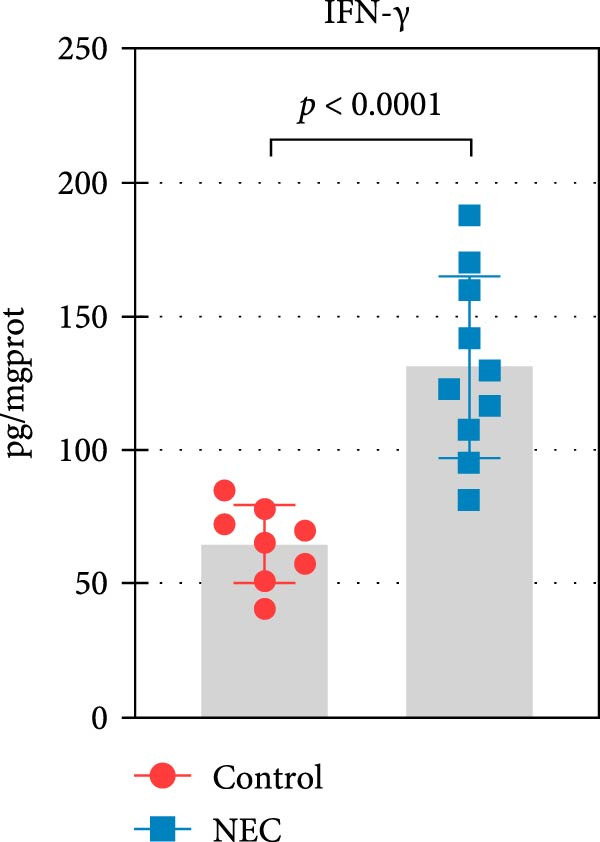
(g)

### 3.3. ALAS2 Expression Was Downregulated, and the Levels of Oxidative Stress and Ferroptosis Were Upregulated in the NEC Model

Next, the expression of ALAS2 in the NEC model was evaluated. As shown in Figure 3a,b, the relative expression of ALAS2 mRNA and protein level in the intestinal tissues of the NEC group was significantly reduced compared with the control group. The immunofluorescence assays also showed similar results (Figure 3c). The changes in the levels of oxidative stress and ferroptosis in the intestinal tissues of NEC group mice were further examined. Compared with the control group, the increased levels of Fe^2+^ and MDA in the ileum tissue of mice in the NEC group were observed, while decreased levels of SOD (Figure 3d–f). In addition, significant morphological changes of mitochondria were observed in the NEC group by transmission electron microscopy, including smaller mitochondrial shrinkage, increased matrix density, and reduced cristae (Figure 3g). The ACSL4 expression was found to be elevated in the intestinal tissues of mice in the NEC group by western blot assay, whereas FTH1 expression was suppressed (Figure 3h–i). Taken together, ALAS2 expression was downregulated, and the levels of oxidative stress and ferroptosis were upregulated in the NEC model.

Figure 3ALAS2 expression was downregulated, and the levels of oxidative stress and ferroptosis were upregulated in the NEC model. (a,b) The expression of ALAS2 in the intestinal tissues of each group was detected by RT‐qPCR and western blot assays. (c) The expression and localization of ALAS2 in the intestinal tissues were detected by immunofluorescence. Original magnification: × 400; scale bar: 50 μm. (d–f) The contents of Fe^2+^, MDA, and SOD activity in intestinal tissues were detected by the kits. (g) The morphology of mitochondria in the intestinal tissues was observed by electron microscope. (h,i) Western blot analysis of ACSL4 and FTH1 expression in the intestinal tissues of each group. Data are presented as mean ± SD. RT‐qPCR, reverse transcription quantitative PCR.

**Figure:**
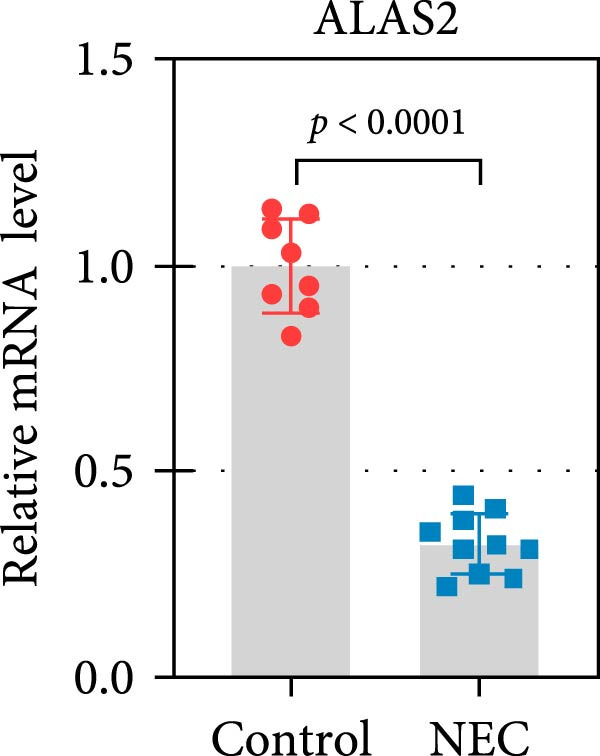
(a)

**Figure:**
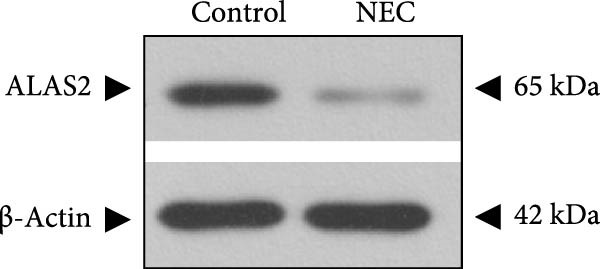
(b)

**Figure:**
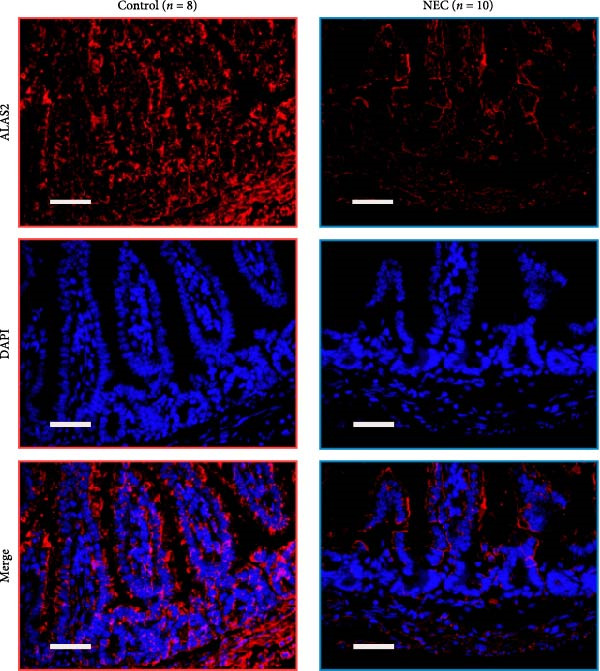
(c)

**Figure:**
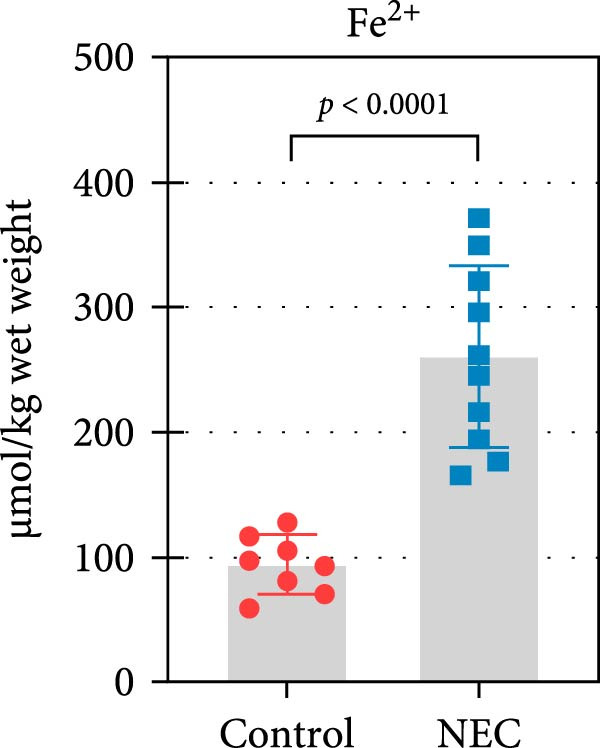
(d)

**Figure:**
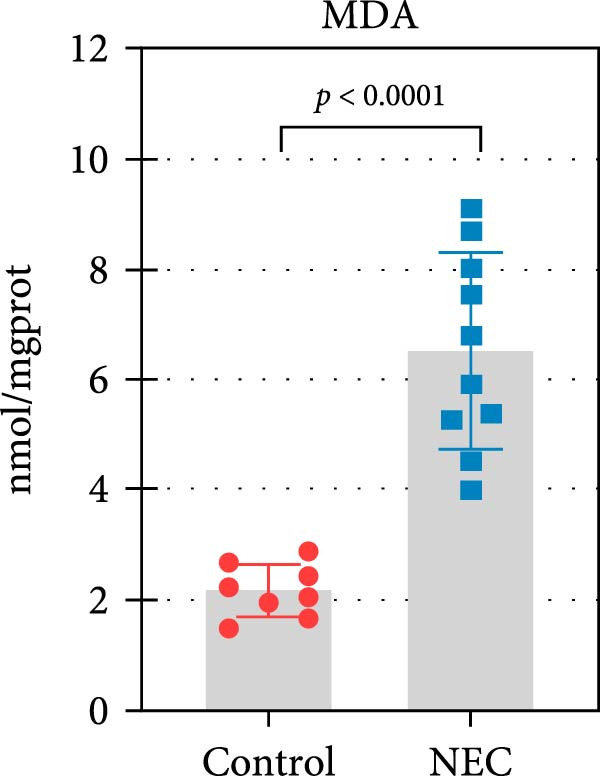
(e)

**Figure:**
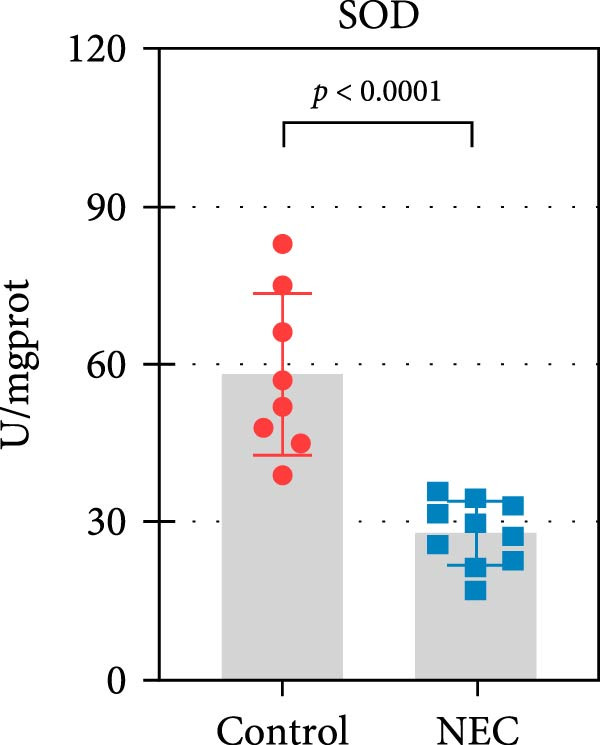
(f)

**Figure:**
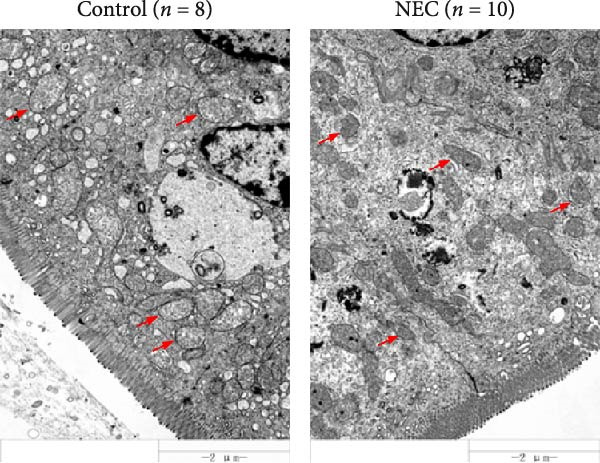
(g)

**Figure:**
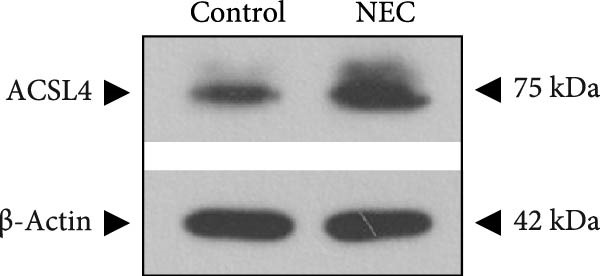
(h)

**Figure:**
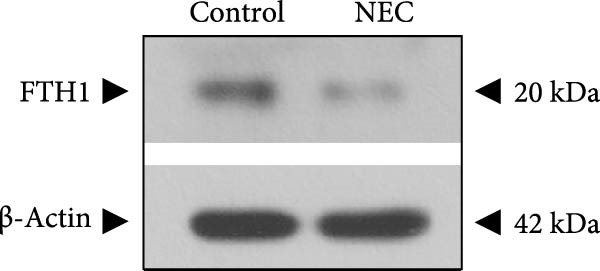
(i)

### 3.4. ALAS2 Expression in IEC‐6 Cells Treated With TNF‐*α* and IFN‐*γ*

The expression and function of ALAS2 in vitro were further explored by inducing IEC‐6 with TNF‐α and IFN‐γ. The cell death at different time points was detected by flow cytometry. As shown in Figure 4a, cell necrosis increased significantly with the extension of treatment time. In addition, the ALAS2 expression was progressively downregulated during cell injury (Figure 4b). Overall, these data suggested that ALAS2 expression was decreased in TNF‐α and IFN‐γ‐induced IECs.

Figure 4ALAS2 expression in IEC‐6 cells treated with TNF‐α and IFN‐γ. (a) Intestinal epithelial cell IEC‐6 was treated with 400 ng/mL TNF‐α and 400 ng/mL IFN‐γ for 0 h, 4 h, 8 h, and 12 h, and the cell necrosis was detected by flow cytometry. (b) The expression of ALAS2 in cells was detected by RT‐qPCR. Data are presented as mean ± SD. RT‐qPCR, reverse transcription quantitative PCR.

**Figure:**
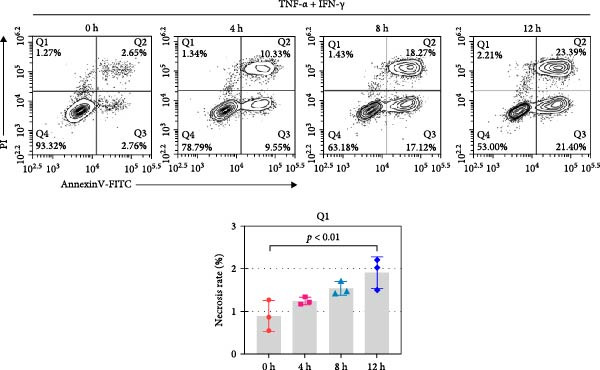
(a)

**Figure:**
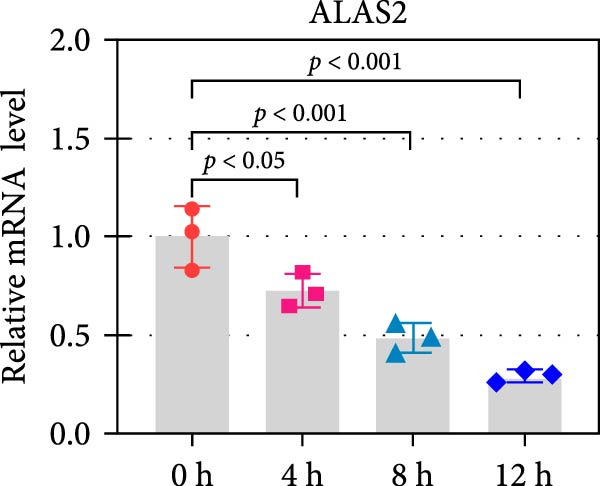
(b)

### 3.5. ALAS2 Inhibits Levels of Oxidative Stress and Ferroptosis in IEC‐6 Cells

The above findings suggested that ALAS2 may play an important role in the progression of NEC. To investigate this hypothesis, the ALAS2 overexpressing IEC‐6 cells was constructed. As shown in Figure 5a,b, the ALAS2 expression was significantly increased in cells transfected with ALAS2 overexpression lentivirus. Then, the effect of ALAS2 overexpression on IEC‐6 cell function was comprehensively evaluated. Treatment with TNF‐α and IFN‐γ resulted in increased cell mortality and inhibited cell viability of cells, but ALAS2 overexpression partially reversed these phenomena (Figure 5c,d). In addition, TNF‐α and IFN‐γ induced increased levels of Fe^2+^ in the cells, suggesting that ferroptosis occurred in the cells (Figure 5e). The lipid ROS levels were detected by the BODIPY C11 probe. As shown in Figure 5f, lipid ROS levels in cells increased after TNF‐α and IFN‐γ exposure. We further observed a decrease in mitochondrial membrane potential by JC‐1 staining in TNF‐α and IFN‐γ‐treated cells (Figure 5g). However, ALAS2 overexpression dulled all of these responses. Subsequently, the MDA content in the cells was examined by the kit and found that ALAS2 overexpression inhibited the increase in MDA levels caused by TNF‐α and IFN‐γ treatment (Figure 5h). In addition, levels of antioxidant stress‐related markers including GSH, CAT, and SOD were downregulated after exposure to TNF‐α and IFN‐γ, but overexpression of ALAS2 significantly restored the expression of these factors (Figure 5i–k). Finally, we examined the expression of ACSL4 and FTH1 in each group of cells by western blot. Unlike ACSLA, the protein expression of FTH1 was reduced in TNF‐α and IFN‐γ induced cells, but the overexpression of ALAS2 partially restored the expression of FTH1 (Figure 5l). Taken together, these results indicated that ALAS2 inhibited levels of oxidative stress and ferroptosis in IEC‐6 cells.

Figure 5ALAS2 inhibits levels of oxidative stress and ferroptosis in IEC‐6 cells. (a,b) The lentiviral vector of ALAS2 overexpression was constructed and packaged to produce lentiviral particles. The infection efficiency was detected by RT‐qPCR and western blot 72 h after the virus infected IEC‐6 cells. (c) The cell necrosis in each group was detected by flow cytometry. (d) Cell viability of respective cells was assessed with CCK‐8 assay. (e) The content of Fe^2+^ was detected by the kit. (f) The lipid ROS content was measured by the BODIPY C11 kit. (g) The changes in mitochondrial membrane potential were detected by JC‐1 staining. Original magnification: × 200; scale bar: 100 μm. (h–k) The contents of MDA, GSH, CAT, and SOD activity in cells were detected by the kits. (l) Western blot analysis of ACSL4 and FTH1 expression in respective cells. Data are presented as mean ± SD. RT‐qPCR, reverse transcription quantitative PCR.

**Figure:**
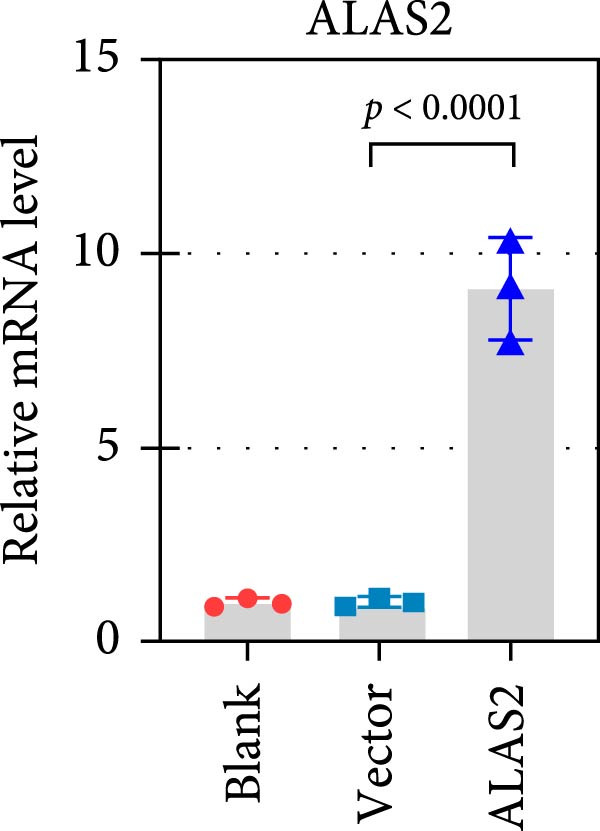
(a)

**Figure:**
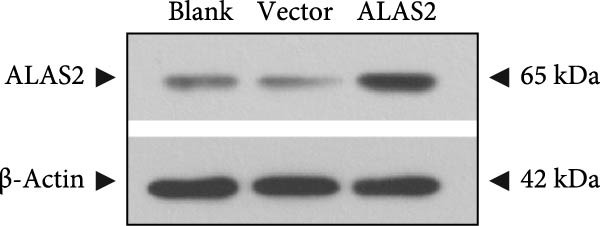
(b)

**Figure:**
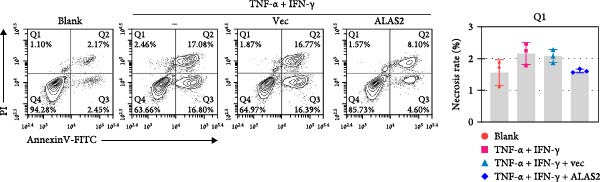
(c)

**Figure:**
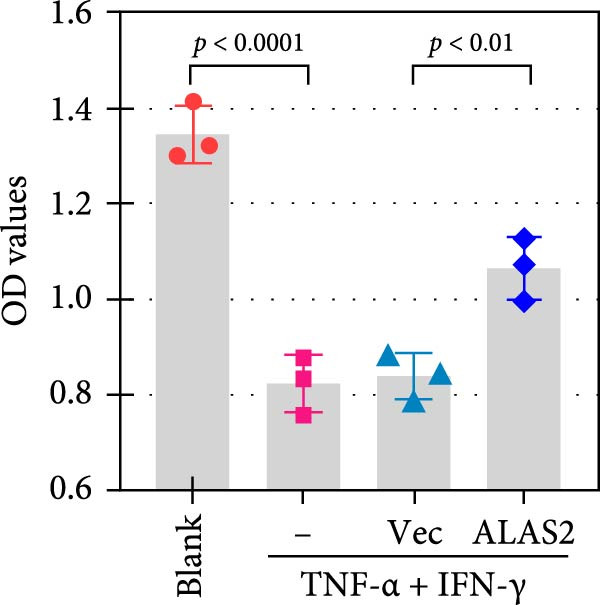
(d)

**Figure:**
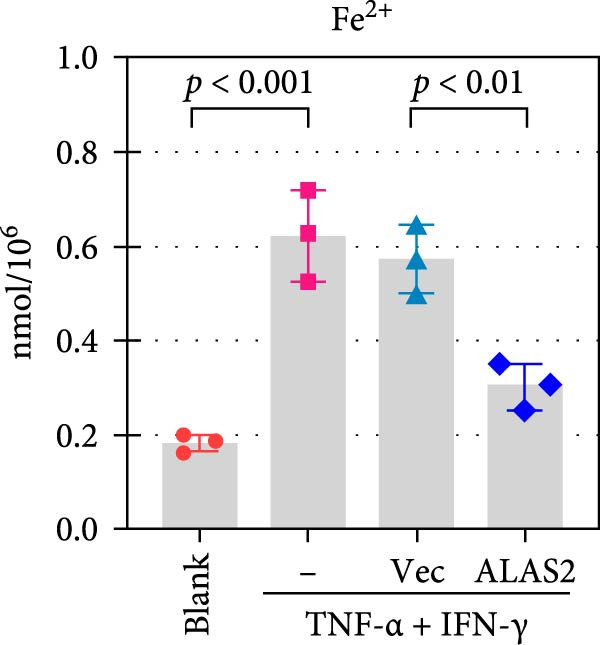
(e)

**Figure:**
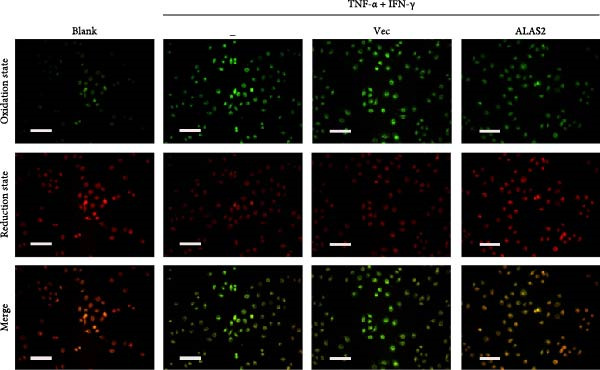
(f)

**Figure:**
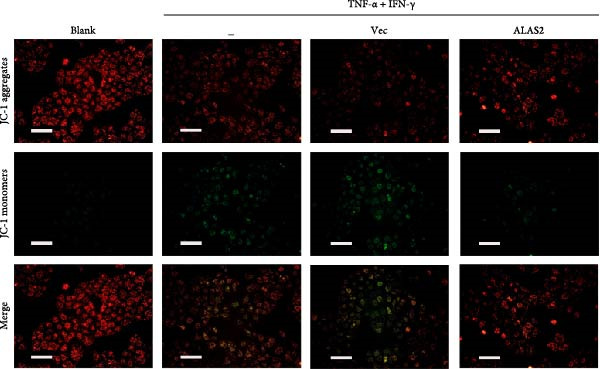
(g)

**Figure:**
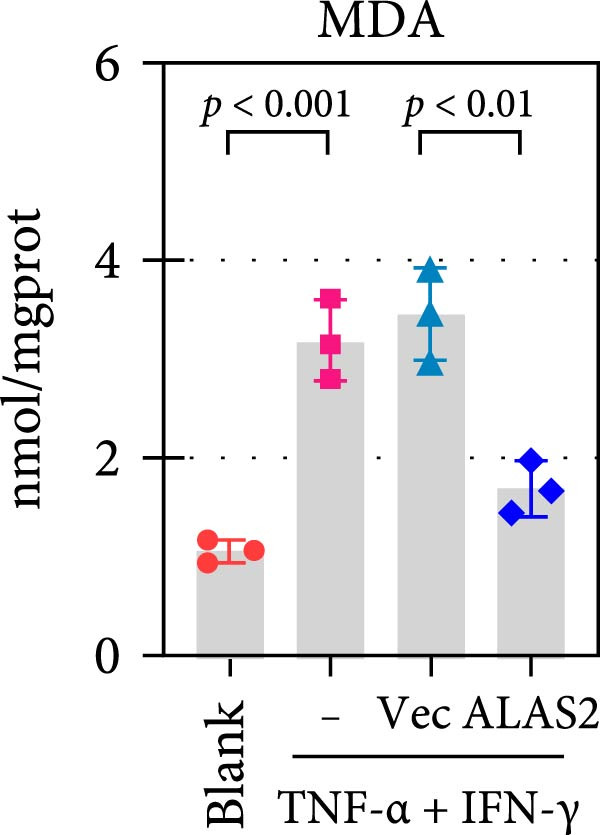
(h)

**Figure:**
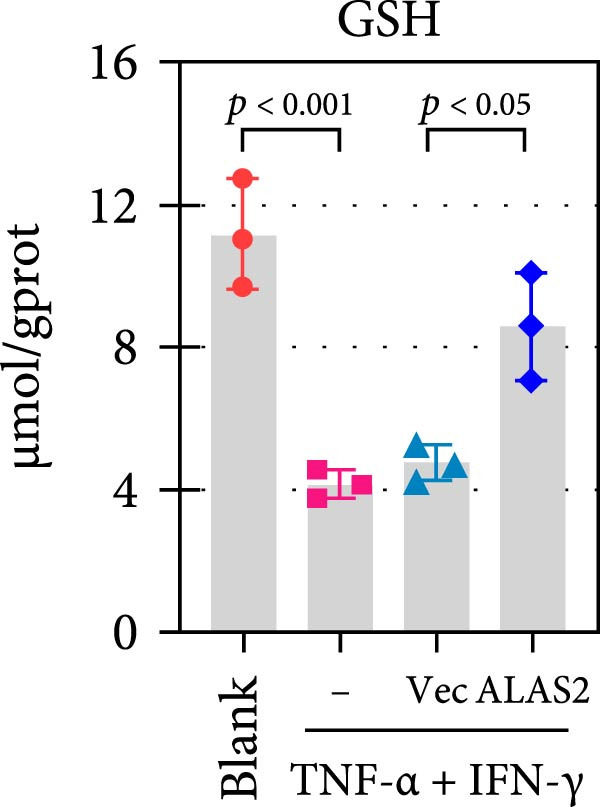
(i)

**Figure:**
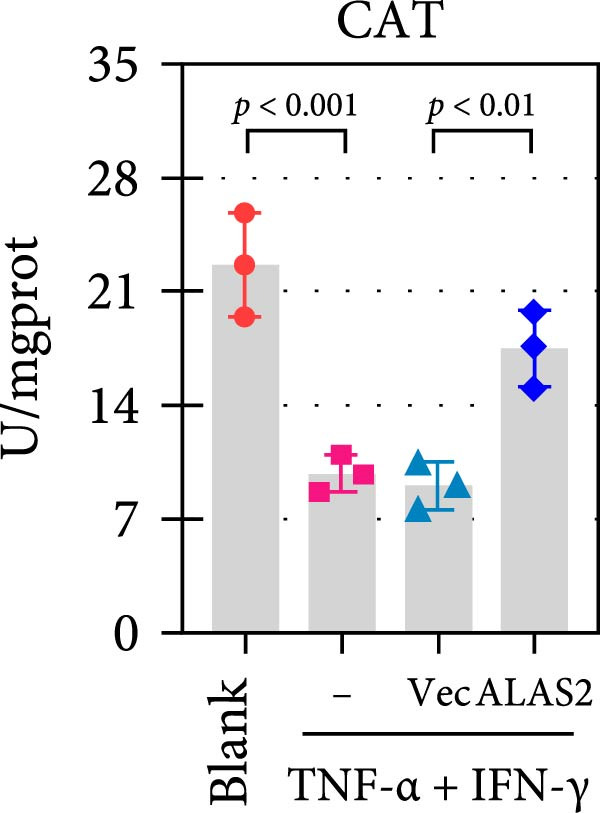
(j)

**Figure:**
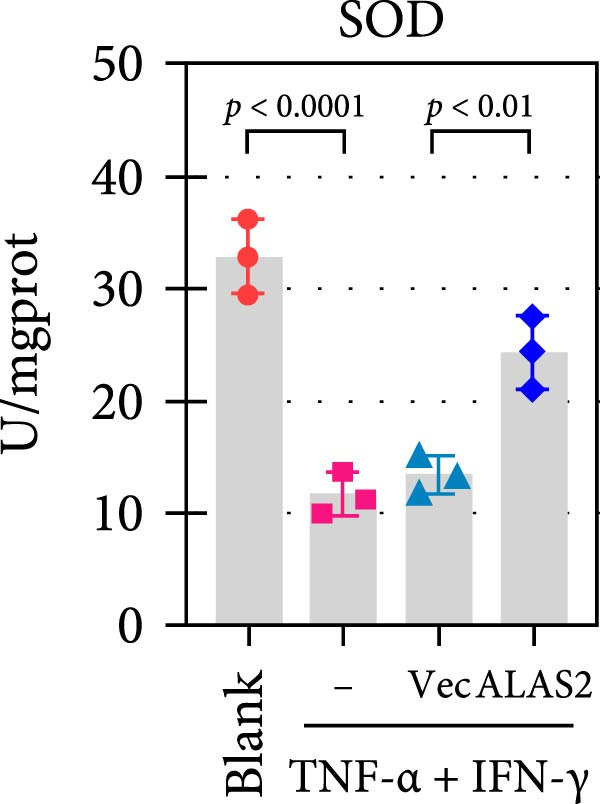
(k)

**Figure:**
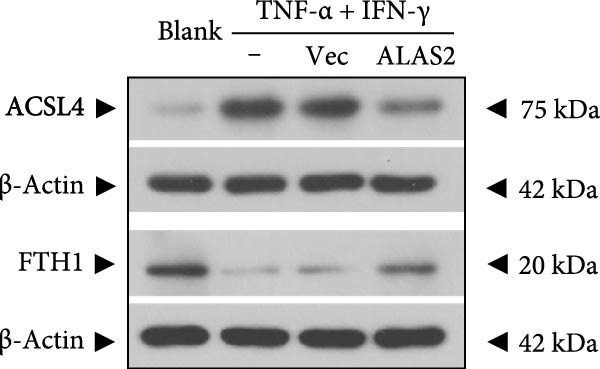
(l)

### 3.6. Untargeted Metabolomics Analysis in IECs With ALAS2 Overexpression

The role of ALAS2 in the progression of NEC was revealed, but the specific mechanism needed to be further studied. Therefore, we collected the control and ALAS2 overexpressing cells and used untargeted metabolomics to explore the metabolites potentially affected by ALAS2. First, the principal component analysis (PCA) was applied to check the overall distribution of the sample data, as shown in Figure 6a. The score chart showed that the samples were scattered into different regions. In addition, supervised orthogonal partial least square discrimination analysis (OPLS‐DA) further demonstrated a good separation pattern between control cells and ALAS2 overexpressing cells. We then used VIP > 1 and *p*  < 0.05 as screening criteria to screen differential metabolites, and the heatmap revealed distinct patterns of metabolites in the control group and the ALAS2 overexpression group (Figure 6b and Table S4). The KEGG pathway enrichment analysis based on altered metabolites revealed significant changes in about 20 biological pathways primarily associated with protein digestion and absorption, pyrimidine metabolism, vitamin digestion and absorption, central carbon metabolism in cancer, biosynthesis of plant secondary metabolites, and metabolic pathway (Figure 6c).

Figure 6Untargeted metabolomics analysis in intestinal epithelial cells with ALAS2 overexpression. Cells were infected with ALAS2 overexpression lentivirus cells for 72 h, then treated with TNF‐α and IFN‐γ for 8 h, and finally were collected for untargeted metabolomics analysis. (a) Score plot of PCA and OPLS‐DA for differential metabolites analysis of ALAS2 overexpression samples and control samples. (b) Clustering heatmap of significantly changed metabolites with *p*  < 0.05 and VIP >1. (c) The KEGG enrichment analysis was performed of differentially expressed metabolites. KEGG, Kyoto Encyclopedia of Genes and Genomes; OPLS‐DA, orthogonal partial least square discrimination analysis; PCA, principal component analysis.

**Figure:**
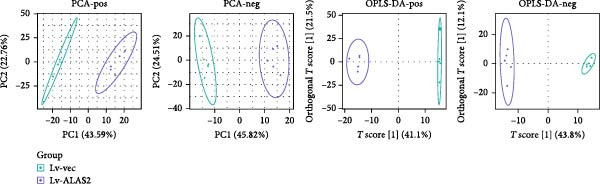
(a)

**Figure:**
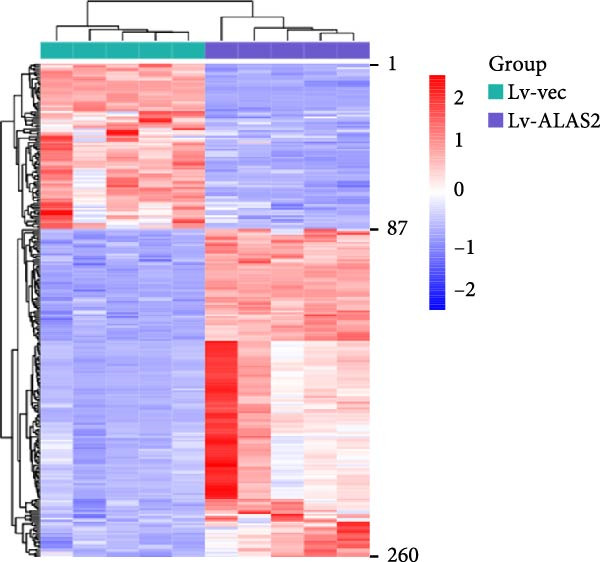
(b)

**Figure:**
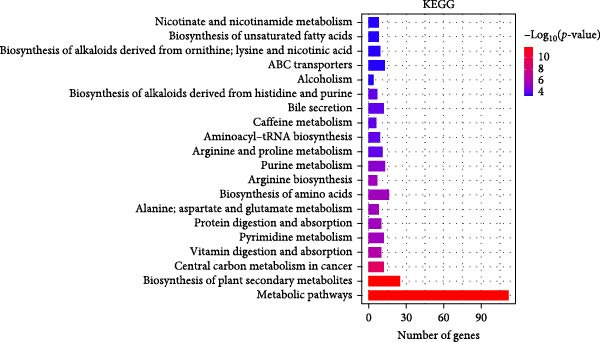
(c)

## 4. Discussion

Despite decades of research, the pathogenesis of NEC remains incompletely understood. Current evidence implicates multifactorial interactions among inflammation, bacterial infection, hypoxia, and other factors [26, 27], with oxidative stress serving as a critical amplifier of injury [28]. Currently, the available treatment options for NEC are not specifically targeted and are mainly focused on improving the intestinal environment [29]. Due to limitations in prevention and treatment, the mortality of NEC has not been significantly improved for a long time. Our study identified that downregulation of ALAS2 is a previously unrecognized contributor to NEC pathogenesis, mechanistically linking impaired heme biosynthesis to oxidative stress and ferroptosis—a finding with significant therapeutic implications.

The centrality of inflammation in NEC is well‐established. Elevated TNF‐α and IFN‐γ perpetuate epithelial barrier disruption through tight junction modulation, causing further deterioration of intestinal barrier function [8, 9]. Consistent with previous reports, the levels of inflammatory factors such as TNF‐α and IFN‐γ were significantly elevated in our NEC mouse model. Inflammatory mediators synergize with oxidative stress, which is particularly detrimental in preterm infants with immature antioxidant defenses [12]. These processes further lead to peroxidation of proteins and cell membranes, as well as excessive MDA production, resulting in cellular damage [30]. Our data confirmed this interaction: NEC tissues exhibited classic oxidative damage markers (increased Fe^2+^/MDA, decreased SOD). Crucially, we extend prior observations of redox imbalance [11, 28] by demonstrating that ALAS2 restoration normalizes antioxidant capacity (increased GSH/CAT/SOD), suggesting a regulatory role beyond its canonical heme synthesis function.

In recent years, the mechanism of intestinal barrier dysfunction caused by excessive death of IECs has been a hot topic in the study of NEC pathogenesis. IECs are continuous monolayer cell structures located in the intestinal lumen, with tight intercellular junctions reinforcing the physical barrier of the intestinal tract and acting as the first defense against environmental and microbial attack [31]. Ferroptosis, an iron‐dependent form of RCD, emerges as a critical mediator of IEC death in NEC [32]. Unlike apoptosis or pyroptosis, ferroptosis is driven by lipid peroxidation and mitochondrial dysfunction, processes tightly linked to oxidative stress [33, 34]. Our findings were consistent with previous reports of free iron and mitochondrial dysfunction observed in NEC models [35]. In our NEC model, IECs exhibited hallmark ferroptotic features: Fe^2+^ accumulation, lipid ROS elevation, mitochondrial shrinkage, upregulated ACSL4 (a ferroptosis promoter), and downregulated FTH1 (an iron‐sequestering protein). These changes directly contributed to IEC death, as evidenced by increased necrosis in vitro. Importantly, overexpression of ALAS2 significantly ameliorated these phenomena, suggesting that ALAS2 could be used as a ferroptosis inhibitor to minimize the degree of intestinal damage in NEC.

Our untargeted metabolomics analysis revealed that ALAS2 overexpression significantly altered metabolic pathways in IECs, including protein digestion and absorption, pyrimidine metabolism, vitamin digestion and absorption, central carbon metabolism in cancer, biosynthesis of plant secondary metabolites, and general metabolic pathways. These pathways are integral to nutrient utilization, nucleic acid synthesis, and redox balance—processes essential for maintaining IEC function and resisting cell death [36, 37]. The observed modulation of vitamin digestion and absorption pathways is particularly noteworthy, as vitamins play crucial roles in antioxidant defense systems [38]. Similarly, the changes in central carbon metabolism may affect cellular energy production and redox balance [39]. The metabolic pathway analysis complements our findings on ALAS2’s ability to reduce oxidative stress and inhibit ferroptosis, offering new insights into potential therapeutic targets for NEC treatment.

While this study identifies ALAS2 as a critical regulator of NEC, several limitations should be acknowledged. First, the mechanistic link between ALAS2 and ferroptosis remains incompletely defined. Although our data demonstrate that ALAS2 overexpression reduces ferroptotic markers, the specific molecular pathways by which ALAS2 modulates iron metabolism and lipid peroxidation require further exploration. Second, our study mainly focused primarily on ferroptosis as the key mode of IEC death, but flow cytometry data indicated that ALAS2 reduces necrosis, and the potential crosstalk between ALAS2 and these alternative cell death pathways was not investigated. Third, untargeted metabolomics identified perturbations in pathways such as protein digestion and pyrimidine metabolism, but the specific metabolites and their functional relevance to ALAS2‐mediated protection remain unclear. Follow‐up studies quantifying key metabolites and validating their roles in antioxidant defense or ferroptosis inhibition are needed to strengthen the metabolic insights. These limitations will be thoroughly analyzed in our subsequent research.

**Figure 7:**
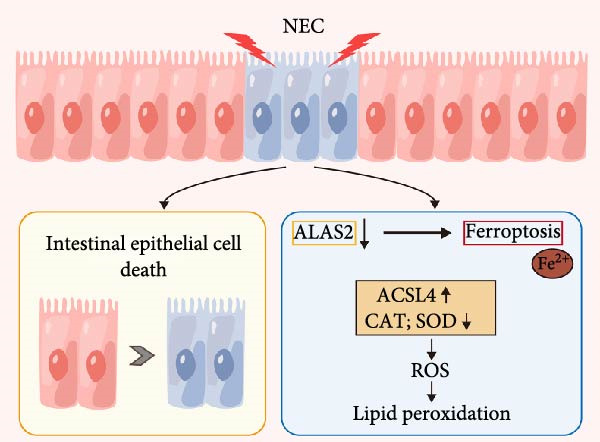
Schematic diagram of the role of ALAS2 in NEC progression. ALAS2 promotes intestinal epithelial cell death and thus aggravates the progression of NEC, which is related to the promotion of oxidative stress and ferroptosis in intestinal epithelial cells.

In summary, this study highlights ALAS2 as a novel protector against NEC, with its downregulation exacerbating IEC death through oxidative stress and ferroptosis, while its overexpression preserves IEC viability via metabolic reprogramming and antioxidant defense (Figure 7). These findings underscore the importance of targeting ALAS2 to maintain IEC survival and intestinal barrier integrity, offering a promising therapeutic strategy for NEC.

## Ethics Statement

All procedures involving animal experiments were performed with consent from the Ethics Committee of the Sixth Affiliated Hospital of Harbin Medical University (Approval Number LC2024‐078).

## Disclosure

All authors read and approved the final manuscript.

## Conflicts of Interest

The authors declare no conflicts of interest.

## Author Contributions

**Zenghui Hao:** conceptualization, methodology, writing – original draft. **Jinbao Han and Ting Yao:** data curation. **Zheng Zhao and Wei Fan:** visualization, investigation. **Zaiqun Jiang and Yunting Wang:** resources, supervision. **Xiaoqian Yang:** software, validation. **Zhilin Xu:** visualization, writing – review and editing. Zenghui Hao and Jinbao Han contributed equally to this work and are designated as co‐first authors.

## Funding

The authors did not receive support from any organization for the submitted work.

## Supporting Information

Additional supporting information can be found online in the Supporting Information section.

## Supporting information


**Supporting Information** Table S1: The list of differentially expressed genes in the GSE198372 database. Table S2: The list of differentially expressed genes in the GSE193177 database. Table S3: The list of differentially expressed genes in the GSE64801 database. Table S4: The list of differential metabolites of Figure 6b.

## Data Availability

All other data that support the findings of this study are available from the corresponding author upon reasonable request.
