# Supplementary material for: ALAS2 Prevents Neonatal Necrotizing Enterocolitis by Improving Ferroptosis in Intestinal Epithelial Cells Through Inhibition of Oxidative Stress
Source: Mediators Inflamm. 2026 Jan 7;2026:6683001. doi: 10.1155/mi/6683001 (PMC12775833; doi:10.1155/mi/6683001)
Supplement: Supplementary file 1 — Supporting Information Table S1: The list of differentially expressed genes in the GSE198372 database. Table S2: The list of differentially expressed genes in the GSE193177 database. Table S3: The list of differentially expressed genes in the GSE64801 database. Table S4: The list of differential metabolites of Figure 6b. [file MI-2026-6683001-s001.zip › Supplementary materials.docx]

Supplementary Table 1. The list of differentially expressed genes in the GSE198372 database.

| Number | Gene | Number | Gene | Number | Gene |
| --- | --- | --- | --- | --- | --- |
| 1 | Fam162a | 41 | Ppm1j | 81 | Ccl11 |
| 2 | Mt1 | 42 | Entpd7 | 82 | Tmem119 |
| 3 | Mt2 | 43 | Btnl2 | 83 | Dio3os |
| 4 | Slc52a3 | 44 | H2bc6 | 84 | Cpxm1 |
| 5 | Tm6sf2 | 45 | Vnn1 | 85 | Slitrk6 |
| 6 | Lrrc4 | 46 | Acot12 | 86 | Flrt1 |
| 7 | Ugt1a9 | 47 | Cbr3 | 87 | Piwil2 |
| 8 | Fabp2 | 48 | Leap2 | 88 | H2bu1−ps |
| 9 | Gk | 49 | 1810055G02Rik | 89 | Car8 |
| 10 | Ephx2 | 50 | Slc47a1 | 90 | Gm12092 |
| 11 | Aldh3b1 | 51 | Paqr7 | 91 | Hoxb9 |
| 12 | Adap1 | 52 | Gstm3 | 92 | Hapln1 |
| 13 | Enpp3 | 53 | Moap1 | 93 | AAdacl4fm3 |
| 14 | Aqp11 | 54 | Slc5a4b | 94 | Bmp3 |
| 15 | Apoa4 | 55 | Gm5485 | 95 | Cyp11a1 |
| 16 | Tkfc | 56 | Slc2a2 | 96 | Il18r1 |
| 17 | Cgref1 | 57 | Slc22a4 | 97 | Slc11a2 |
| 18 | Mttp | 58 | Gsta2 | 98 | Trbd1 |
| 19 | Lct | 59 | Lpar5 | 99 | Ube2l3 |
| 20 | Mmp15 | 60 | Ftl1−ps2 | 100 | Mir196a−1 |
| 21 | Ceacam18 | 61 | Acaa1b | 101 | Mex3a |
| 22 | Igsf23 | 62 | Gm3776 | 102 | Timp3 |
| 23 | Me1 | 63 | Abcb1a | 103 | Lgr5 |
| 24 | Gm7049 | 64 | Gsta1 | 104 | Cspg4b |
| 25 | Sis | 65 | Nqo1 | 105 | Sgpp1 |
| 26 | Fabp1 | 66 | Pappa2 | 106 | Afm |
| 27 | Gclc | 67 | Cfi | 107 | Ckmt2 |
| 28 | Acta1 | 68 | Enpp7 | 108 | H60b |
| 29 | Gpd2 | 69 | Fmo5 | 109 | Anxa8 |
| 30 | Pdk4 | 70 | Gstm1 | 110 | Afp |
| 31 | H2−Q1 | 71 | 2010106E10Rik | 111 | Alas2 |
| 32 | Gata4 | 72 | Apoc2 | 112 | Slc6a14 |
| 33 | Por | 73 | Gm8834 | 113 | Hbb−bt |
| 34 | Gda | 74 | H19 | 114 | Coq8a |
| 35 | Cidec | 75 | Serpina1c | 115 | Hoxb6 |
| 36 | Rbp2 | 76 | Lrp2 | 116 | Hoxb5 |
| 37 | Creb3l3 | 77 | Mirlet7a−2 |  |  |
| 38 | Hkdc1 | 78 | Hoxb7 |  |  |
| 39 | C2cd2 | 79 | Eva1a |  |  |
| 40 | Slc5a1 | 80 | Gal3st2 |  |  |

Supplementary Table 2. The list of differentially expressed genes in the GSE193177 database.

| Number | Gene | Number | Gene | Number | Gene |
| --- | --- | --- | --- | --- | --- |
| 1 | Car3 | 41 | Clca4a | 81 | Gpt |
| 2 | Ighm | 42 | Gsta1 | 82 | Fabp2 |
| 3 | Cd79b | 43 | Gml2 | 83 | Slc7a15 |
| 4 | Enpp2 | 44 | Gm3776 | 84 | Enpp3 |
| 5 | Ccn3 | 45 | Trim40 | 85 | H2-Q1 |
| 6 | Scara3 | 46 | Abcb1a | 86 | Apoc2 |
| 7 | Itm2a | 47 | Mt1 | 87 | Fos |
| 8 | Igfbp5 | 48 | Mt2 | 88 | Mmp10 |
| 9 | Alas2 | 49 | Gsta2 | 89 | Gml |
| 10 | Hbb-bt | 50 | Tmigd1 | 90 | Fabp1 |
| 11 | Hbb-b1 | 51 | Slc51b | 91 | 5033404E19Rik |
| 12 | Hba-a1 | 52 | 2010106E10Rik | 92 | Eif4ebp3 |
| 13 | Hba-a2 | 53 | Gk | 93 | Nat8f5 |
| 14 | Slit2 | 54 | Vnn1 | 94 | 1700016C15Rik |
| 15 | Nr1d1 | 55 | Cyp4f14 | 95 | Fabp6 |
| 16 | Mdk | 56 | Akr1c19 | 96 | Lrrc26 |
| 17 | Nrep | 57 | Sult2b1 | 97 | Saa3 |
| 18 | Olfml1 | 58 | Gstm3 | 98 | Gm10036 |
| 19 | Fkbp1b | 59 | Scin | 99 | Defa43 |
| 20 | Ager | 60 | Sis | 100 | Ccl20 |
| 21 | Aoah | 61 | Krt20 | 101 | Defa34 |
| 22 | Ceacam2 | 62 | Slc10a2 | 102 | Mptx1 |
| 23 | Ifit3 | 63 | Sult6b2 | 103 | Spink4 |
| 24 | Ifit1 | 64 | Mgat4c | 104 | Mmp7 |
| 25 | Kng2 | 65 | Gpx2 | 105 | Mptx2 |
| 26 | Ifit1bl1 | 66 | Nabp1 | 106 | Defa22 |
| 27 | H2-Q10 | 67 | Prss32 | 107 | Defa41 |
| 28 | Notum | 68 | Pglyrp1 | 108 | Itln1 |
| 29 | Sapcd2 | 69 | Dmbt1 | 109 | Defa24 |
| 30 | Lect2 | 70 | Fmo5 | 110 | Defa32 |
| 31 | Chac1 | 71 | Clca1 | 111 | Defa30 |
| 32 | Scd2 | 72 | Sprr2a3 | 112 | Defa2 |
| 33 | Trib3 | 73 | Sprr2a2 | 113 | Defa21 |
| 34 | H60b | 74 | Sprr2a1 | 114 | Defa3 |
| 35 | Afp | 75 | Noxa1 | 115 | Defa33 |
| 36 | Slc11a2 | 76 | Pigr | 116 | Klk1 |
| 37 | Anxa8 | 77 | Reg3g | 117 | Defa23 |
| 38 | Xlr3b | 78 | Agr2 | 118 | Defa20 |
| 39 | Acta1 | 79 | Reg3b | 119 | Defa5 |
| 40 | Rab30 | 80 | Barx2 |  |  |

Supplementary Table 3. The list of differentially expressed genes in the GSE64801 database.

| Number | Gene |
| --- | --- |
| 1 | TNNT3 |
| 2 | ALAS2 |
| 3 | SLC4A1 |
| 4 | HEMGN |
| 5 | HBE1 |
| 6 | EPB42 |
| 7 | GYPA |
| 8 | JCHAIN |
| 9 | NOS2 |
| 10 | REG1A |

Supplementary Table 4. The list of differential metabolites of Fig. 6b.

| Number | Name | Number | Name | Number | Name |
| --- | --- | --- | --- | --- | --- |
| 1 | Betaine | 88 | D-Glucosamine 6-phosphate | 175 | PC 8:0_9:0 |
| 2 | 4-oxododecanedioic acid | 89 | Ala-Gln | 176 | CAR 20:0 |
| 3 | L-Methionine sulfone | 90 | CAR 20:4 | 177 | all-cis-4,7,10,13,16-Docosapentaenoic acid |
| 4 | 5-Methyluridine | 91 | 2'-Deoxyadenosine-5'-monophosphate | 178 | Oleoyl ethanolamide |
| 5 | NMK | 92 | Prostaglandin E2 | 179 | RLH |
| 6 | 2-(2-amino-3-methylbutanamido)-3-phenylpropanoic acid | 93 | dAMP | 180 | LPS 18:1 |
| 7 | 4-Hydroxybenzoic acid | 94 | 1,5,8-Trihydroxy-9-oxo-9H-xanthen-3-yl beta-D-glucopyranoside | 181 | SM 15:3;2O/22:6 |
| 8 | Kynurenic acid | 95 | Ala-trp | 182 | LPI 20:4 |
| 9 | L-Methionine Methyl Ester | 96 | 2-hydroxy-6-[(8Z,11Z)-pentadeca-8,11,14-trien-1-yl]benzoic acid | 183 | LNAPE 20:4/N-3:0 |
| 10 | L-beta-Imidazolelactic acid | 97 | Prostaglandin A1 ethyl ester | 184 | LPC O-18:2 |
| 11 | Cytosine | 98 | Chenodeoxycholic Acid | 185 | Docosapentaenoic acid |
| 12 | Cytidine | 99 | Salvinorin A | 186 | Docosahexaenoic acid |
| 13 | DL-Arginine | 100 | LPG 20:5 | 187 | Arachidonic acid |
| 14 | L(-)-Carnitine | 101 | Trovafloxacin | 188 | (+/-)11(12)-DiHET |
| 15 | Benzyl cinnamate | 102 | Thymidine 5'-monophosphate | 189 | Diosgenin |
| 16 | L-arginine | 103 | Thymidine 5'-diphosphate | 190 | TG 15:1_18:3_18:3 |
| 17 | LPC 14:1-SN1 | 104 | LPS 20:4 | 191 | RNK |
| 18 | 4-Guanidinobutyric acid | 105 | Orotic Acid | 192 | 2-(14,15-Epoxyeicosatrienoyl) glycerol |
| 19 | 2-[(3S)-1-(3,4-Difluorobenzyl)-3-pyrrolidinyl]-1,3-benzothiazole | 106 | 2,4-Dimethylbenzaldehyde | 193 | 17(S)-HpDHA |
| 20 | Vatalanib dihydrochloride | 107 | Nicotinic acid | 194 | LPG 22:5 |
| 21 | L-(+)-Arginine | 108 | Cyclohexylsulfamate | 195 | LPG 22:4 |
| 22 | L-Hydroxyproline | 109 | 3-Methylxanthine | 196 | Glycerol 1-hexadecanoate |
| 23 | 8-Aminooctanoic acid | 110 | Pseudouridine | 197 | mesaconic acid |
| 24 | 3-Hydroxy-3-methylbutanoic acid | 111 | gamma-Glutamyltyrosine | 198 | LPE O-18:1 |
| 25 | Nicotinamide | 112 | Orotidine 5'-monophosphate | 199 | LPC 22:3-SN1 |
| 26 | N-[4-(4-methylpiperazino)phenyl]-2-phenylacetamide | 113 | Asp-Phe methyl ester | 200 | LPC O-16:0 |
| 27 | Desmethylclozapine | 114 | N,5-Bis(3-(trifluoromethyl)phenyl)oxazol-2-amine | 201 | 7-Ketocholesterol |
| 28 | Asaraldehyde | 115 | AICA ribonucleotide | 202 | Lysops 22:6 |
| 29 | Dimetghyl 4-Hydroxyisophthalate | 116 | Thymine | 203 | Lysopg 18:1 |
| 30 | Phosphopyruvic acid | 117 | N1-{3-[2-(2-pyridyl)ethyl]phenyl}-4-chlorobenzene-1-sulfonamide | 204 | Stearic acid |
| 31 | 3-(2-thienylmethylidene)chroman-4-one | 118 | N-(4-chlorophenethyl)-N'-(4-chlorophenyl)urea | 205 | (±)8(9)-DiHET |
| 32 | (2R,3S,4S,5R,6R)-2-(hydroxymethyl)-6-(2-phenylethoxy)oxane-3,4,5-triol | 119 | Cyclocytidine | 206 | 1-Palmitoylglycerol |
| 33 | 2-Methylbutyroylcarnitine | 120 | 1-Methylxanthine | 207 | All-Trans-13,14-Dihydroretinol |
| 34 | DY131 | 121 | D-(-)-Mannitol | 208 | octadec-9-ynoic acid |
| 35 | α-Ergocryptine | 122 | Pyridoxine | 209 | Tomatidine |
| 36 | Methyl Violet | 123 | 2-Phenylglycine | 210 | 1-Stearoylglycerol |
| 37 | 2-(2-oxo-2-{[2-(2-oxo-1-imidazolidinyl)ethyl]amino}ethoxy)acetic acid | 124 | methyl 3-[(isoxazol-5-ylcarbonyl)amino]thiophene-2-carboxylate | 211 | 11(Z),14(Z),17(Z)-Eicosatrienoic acid |
| 38 | Secnidazole | 125 | 5'-Deoxy-5'-(Methylthio)Adenosine | 212 | SM 15:3;2O/18:5 |
| 39 | D-Ribose-1-phosphate | 126 | LPI 20:5 | 213 | Epitestosterone glucuronide |
| 40 | UR-144 N-(5-hydroxypentyl) β-D-glucuronide | 127 | N6-Succinyl Adenosine | 214 | N-Acetylsphingosine |
| 41 | RQH | 128 | 2'-Deoxyinosine | 215 | FAHFA 16:0/18:2 |
| 42 | Biotin | 129 | 7-Methylxanthine | 216 | LPE 22:3 |
| 43 | N-(1,3-benzodioxol-4-ylmethyl)-6,7-dimethoxyquinazolin-4-amine | 130 | Oxoadipic Acid | 217 | PC O-22:3 |
| 44 | 2-hydroxyiminoindane-1,3-dione | 131 | Isorhapontigenin | 218 | LPE 22:5 |
| 45 | 3-Methoxytyramine | 132 | S-Adenosyl-L-methionine | 219 | LSD-d3 |
| 46 | Guanethidine Monosulfate | 133 | Octopamine | 220 | LPI 22:3 |
| 47 | LysoPE 18:2 | 134 | S-Adenosylmethionine | 221 | PC O-22:4 |
| 48 | Glycerophospho-N-palmitoyl ethanolamine | 135 | Xanthosine | 222 | N'1-[1-(2-hydroxyphenyl)ethylidene]-3-methoxybenzene-1-carbohydrazide |
| 49 | LPC 14:0 | 136 | N,5-Bis(3-nitrophenyl)oxazol-2-amine | 223 | FAHFA 18:1/22:5 |
| 50 | LPC 14:1 | 137 | Melatonin | 224 | 11(E)-Eicosenoic Acid |
| 51 | LPC 16:2-SN1 | 138 | Choline bitartrate | 225 | FAHFA 18:1/20:3 |
| 52 | LPC 12:0 | 139 | S-Adenosylhomocysteine | 226 | Docosatrienoic acid |
| 53 | 12-Hydroxydodecanoic acid | 140 | 2'-Deoxyinosine 5'-monophosphate | 227 | 11(Z),14(Z)-Eicosadienoic Acid |
| 54 | Lysope 14:0 | 141 | L-Arabinitol | 228 | Elaidic acid |
| 55 | D-Xylonic Acid | 142 | Allantoin | 229 | 8Z,11Z,14Z-Eicosatrienoic acid |
| 56 | (R)-3-Hydroxy myristic acid | 143 | 14,15-Leukotriene E4 | 230 | LPG 14:0 |
| 57 | Homogentisic Acid | 144 | Dl-3-Hydroxy-kynurenine | 231 | N-(4-chlorophenyl)-N'-(2-phenoxyphenyl)urea |
| 58 | 1-oxo-2,3-dihydro-1H-inden-4-yl benzoate | 145 | 3-Hydroxy-3-methylglutaric acid | 232 | AKB48 N-(4-hydroxypentyl) metabolite |
| 59 | Formononetin | 146 | D-Homocysteine | 233 | Folic acid |
| 60 | 7-{[(2E)-3,7-dimethylocta-2,6-dien-1-yl]oxy}-2H-chromen-2-one | 147 | Prostaglandin E2-1-glyceryl ester | 234 | 1a,1b-Dihomo prostaglandin E1 |
| 61 | Orotidine | 148 | 2-hexyl-5-(4-nonylphenyl)pyrimidine | 235 | Prostaglandin F2α-1-glyceryl ester |
| 62 | Propionyl-L-carnitine | 149 | LPC 22:4-SN1 | 236 | (2R,3S,4S,5R,6S)-2-(hydroxymethyl)-6-phenoxyoxane-3,4,5-triol |
| 63 | N,N-dibutyl-4-[2-(4-nitrophenyl)diazenyl]aniline | 150 | LPE 22:6 | 237 | Milbemycin A3 oxime |
| 64 | 5-(4-benzylpiperazino)-2,4(1H,3H)-pyrimidinedione | 151 | Adrenic acid | 238 | L-Ornithine |
| 65 | Bisphenol TMC | 152 | RKK | 239 | 5α-Dihydrotestosterone glucuronide |
| 66 | Enrofloxacin | 153 | 2-Linoleoyl glycerol | 240 | PC O-40:9 |
| 67 | 11-dehydro Thromboxane B2 | 154 | 2-Arachidonyl Glycerol ether | 241 | H-Gly-Pro-OH |
| 68 | 2-Methoxyestrone | 155 | LPC O-16:1 | 242 | L-Cysteine-glutathione gisulfide |
| 69 | DL-Carnitine | 156 | LPG 16:0 | 243 | Cystine |
| 70 | Argininosuccinic acid | 157 | 2,4-dihydroxyheptadec-16-en-1-yl acetate | 244 | Cytidine 5'-diphosphocholine |
| 71 | 15-Deoxy-Δ12,14-prostaglandin D2 | 158 | LPC 20:5 | 245 | Aflatoxin G2 |
| 72 | Tretinoin | 159 | LPC 22:6 | 246 | Glycerol-3-phosphate |
| 73 | Maltotriose | 160 | 3-Oxo-7alpha,12alpha-hydroxy-5beta-cholanoic acid | 247 | Aflatoxin B1 |
| 74 | Feruloyl Putrescine | 161 | Lysopa 16:0 | 248 | Bilirubin |
| 75 | L-Leucyl-L-Alanine | 162 | LPC 20:4-SN1 | 249 | UDP-N-acetylglucosamine |
| 76 | LPE 16:2 | 163 | N-Oleoyl Glycine | 250 | Quercetin-3β-D-glucoside |
| 77 | LPC 15:0-SN1 | 164 | LNAPE 18:1/N-3:0 | 251 | 5-[(Benzoyloxy)methyl]-4,5,6-trihydroxy-2-cyclohexen-1-yl benzoate |
| 78 | Tyrosylalanine | 165 | LPE O-18:0 | 252 | 7-hydroxy-6-nitro-2,3-dihydro-1H,5H-pyrido[3,2,1-ij]quinolin-5-one |
| 79 | Guanosine | 166 | PC O-20:5 | 253 | S-(Methyl)Glutathione |
| 80 | Guanine | 167 | LPC 22:5 | 254 | Acetylcarnitine |
| 81 | (±)10(11)-EpDPA | 168 | PC O-20:4 | 255 | N-lactoyl-phenylalanine |
| 82 | 13(S)-HOTrE | 169 | LPC 20:4 | 256 | Nicotinamide N-oxide |
| 83 | CAR 18:1 | 170 | LPC 22:4 | 257 | (3R)-8-hydroxy-3-(4-methoxyphenyl)-3,4-dihydro-1H-2-benzopyran-1-one |
| 84 | SPB 18:1;2O | 171 | PC O-22:5 | 258 | N-(4-chlorophenyl)-N'-[3-methoxy-5-(trifluoromethyl)phenyl]thiourea |
| 85 | Ala-Leu | 172 | PC O-20:3 | 259 | 1-acetyl-N-(6-chloro-1,3-benzothiazol-2-yl)-4-piperidinecarboxamide |
| 86 | Penicillin G | 173 | 2-Arachidonoyl glycerol | 260 | 2-Naphthol |
| 87 | 5-Methoxyindole-3-Carbaldehyde | 174 | Monoolein |  |  |
