# Supplementary material for: ALAS2 Prevents Neonatal Necrotizing Enterocolitis by Improving Ferroptosis in Intestinal Epithelial Cells Through Inhibition of Oxidative Stress
Source: Mediators Inflamm. 2026 Jan 7;2026:6683001. doi: 10.1155/mi/6683001 (PMC12775833; doi:10.1155/mi/6683001)
Supplement: Supplementary file 1 — Supporting Information Table S1: The list of differentially expressed genes in the GSE198372 database. Table S2: The list of differentially expressed genes in the GSE193177 database. Table S3: The list of differentially expressed genes in the GSE64801 database. Table S4: The list of differential metabolites of Figure 6b. [file MI-2026-6683001-s001.zip › Response letter_2.docx]

Response to editor

Dear editors,

Thank you for your email and for providing the valuable feedback regarding our manuscript [6683001], titled “ALAS2 prevents neonatal necrotizing enterocolitis by improving ferroptosis in intestinal epithelial cells through inhibition of oxidative stress.”

We have carefully addressed both points raised in your message:

1. Editable High-Resolution Figures:
All figures have now been uploaded as separate editable PS files (as required) in the “Figure file” section. These files allow full editing of lines, arrowheads, fonts, and other elements to align with the journal’s style.

2. Supplementary Material Description:
A concise description for each supplementary material file has been included in the manuscript file, just before the reference section.

We believe that all requested revisions have been fully addressed. Please do not hesitate to let us know if any further adjustments or additional information are required.

Thank you for your time and assistance. We look forward to the next steps in the publication process.

Yours sincerely,

Zhilin Xu

Department of Pediatric Surgery, The Sixth Affiliated Hospital of Harbin Medical University, Harbin, People's Republic of China

Email: [xzlin333@163.com](mailto:xzlin333@163.com)
